# Effects of chronic estradiol treatment on the thyroid gland structure and function of ovariectomized rats

**DOI:** 10.1186/1756-0500-2-173

**Published:** 2009-08-30

**Authors:** Menna M Abdel-Dayem, Mohamed S Elgendy

**Affiliations:** 1 Histology Department, Faculty of Medicine, Cairo University, Cairo, Egypt; 2 Histology Department, Faculty of Medicine, Fayoum University, Fayoum, Egypt

## Abstract

**Background:**

Estrogen therapy is widely used nowadays in women to treat many postmenopausal symptoms but it may have some undesirable effects due to multiple organs affection. So, the aim of this study was to determine the effects of chronic estradiol treatment on the structure and function of the thyroid gland in ovarictomized rats as a model simulating menopause.

**Findings:**

Thirty adult female Wistar rats divided into three groups were used in this study; the first group was sham-operated, while the second and third groups were ovariectomized. The first and second groups were injected with olive oil while the third group was injected with estradiol dipropionate daily for three months, after that; hormonal assay for T3, T4, TSH and specimens of the thyroid were taken and processed to be examined by light and electron microscopy. The results of this study revealed that serum levels of T3 and T4 decreased in ovariectomized animals and significantly increased after estradiol treatment, while TSH increased in ovariectomized animals and decreased with estradiol treatment. Histological and morphometric study in ovariectomized group revealed marked accumulation of colloid in follicular lumens with decreased epithelial height in addition to increased connective tissue amount. After estradiol treatment the follicles became smaller in size, having small amount of colloid with increased epithelial height in addition to decreased connective tissue content. Ultrastructural study supported these results in addition to the presence of large amount of intracytoplasmic colloid vesicles after estradiol treatment.

**Conclusion:**

Low estrogen level may lead to mild thyroidal hypofunction while estradiol treatment may lead to hyperactivity so it should be used very cautiously in the treatment of postmenopausal symptoms to avoid its undesirable stimulatory effect on the thyroid.

## Background

There is a great wealth of published data on the value of estrogen therapy in alleviating menopausal symptoms such as hot flushes and insomnia and in preventing urogenital atrophy and osteoporosis [1].

During hormonal therapy both beneficial and undesirable side effects appear due to multiple target organs for estrogen [2]. Epidemiological studies suggest that the use of estrogen may contribute to the pathogenesis of thyroid tumors [3] additionally; thyroid diseases are more common in women [4].

Although some studies have demonstrated influences of estrogen on the development, physiology and histology of the thyroid gland, questions about the effects of estrogen replacement therapy on the thyroid gland remained unanswered [5].

As regard the effect of estradiol on thyroid activity; numerous studies have demonstrated contradictory results either stimulatory effect through activation of pituitary-thyroid axis following estradiol treatment [6,7] or no alterations of TSH although decreased T3 in ovariectomized rats which became normal after estradiol treatment [8].

In women treated with conjugated estrogen 0.625-2.5 mg/kg/day, there was no change in serum TSH concentrations [9]. In other studies; perimenopausal and postmenopausal women had "elevated" serum TSH concentrations after 1 month [10] or 6 weeks [11] of estrogen therapy.

Having in mind discrepancies in literature, this study aimed to examine the effects of chronic estradiol treatment on structure and function of thyroid gland in ovarictomized rats as an animal model for menopause.

## Methods

### Animals

Thirty adult female Wistar albino rats of 200 - 220 g body weight were used in this study; they were obtained and housed in the animal house of Kasr-El-Aini Faculty of Medicine, Cairo University, Egypt. The animals received a standard diet for rodents and allowed free access to water. The animals were treated humanely and care was taken to ease suffering.

### Surgical procedure

The animals were divided randomly into three groups of ten animals each. The first group (Group I) was sham-operated. The other two groups were ovariectomized. All animals were anaesthetized with intraperitoneal injection of Phenobarbital sodium (15 mg/kg body weight). The lower abdomen of the rats was shaved and incised. Fallopian tubes and ovaries were identified. Absorbable catgut sutures were used to tie the Fallopian tubes below the ovaries. Then, the ovaries were removed. Sham-operated group underwent a similar surgical incision exposing the ovaries and replacing them in the same position [12].

### Treatment protocol

One month after surgical procedures, rats of the first and second groups were injected intra peritoneal (i.p) with sterile olive oil once daily for three months. The third group was injected (i.p) with 0.625 mg/kg bw. estradiol dipropionate (EDP) (Sigma Chemical Co.), dissolved in sterile olive oil once daily for the same duration. This dose is equivalent to a commonly used dose for estrogen replacement in the clinical practice in postmenopausal women and used in previous experimental designs [13].

This study has been approved by the ethics committee on animal research in the animal house of Kasr-El-Aini Faculty of Medicine, Cairo University, Egypt following international ethics and regulations for animal research in laboratory applications [14].

### Evaluation methods

#### Hormonal assay

At the end of experimental period, blood samples were collected from the tail vein of all animals and centrifuged at 3000 rpm for ten minutes. Sera were separated and stored at -20°C until hormonal assay.

Total T3 and T4 levels were measured by radioimmunoassay (RIA) using commercial kits (Coat-A-Coat), while Serum TSH was measured by RIA using a specific rat TSH kit (supplied by Diagnostic Products Corporation DPC, Los Angeles, USA). Radioactivity was determined by the gamma-counter [15].

The data were presented as mean ± SD.

### Histological study

Twenty four hours after the last injection, all animals were anaesthetized with Phenobarbital sodium, and after intracardiac perfusion with the fixative solution (2% gluteraldehyde in phosphate buffer), the thyroid glands were removed together with a portion of the adjoining trachea. One lobe of the thyroid gland of the animals were dissected, cut into small cubes (about 1 mm^3^) and immediately fixed in 2% gluteraldehyde. for electron microscopic study:

#### Light microscopic study

The remaining lobes of the thyroids were fixed in Bouin's solution and processed for paraffin sections that were stained with Hx&E, Periodic Acid Schiff (PAS) and Masson's trichrome stains.

#### Morphometry

The data were obtained by using "Leica Qwin 500" image analyzer computer system (England). The measurements were done using an objective lens of magnification 40, examining the central regions of the glands. (peripheral and isthmic follicles were excluded).

Epithelial height and follicular area were measured in sections stained with Hx&E. colloidal area percent was assessed in PAS stained sections and connective tissue area percent in sections stained with Masson's trichrome stain. For each parameter, ten readings per animal in each group were done.

#### Electron microscopic study

Small fragments from the thyroid glands were rinsed in phosphate buffer (PH 7.4) fixed in 2% gluteraldehyde, postfixed in 1% osmium tetroxide and dehydrated. After embedding in ultrathin sections were cut and stained with lead citrate and uranyl acetate. The grids were examined and photographed with electron microscope.

### Statistical analysis

The data obtained from hormonal assay and morphometry were presented as mean ± SD. Data analysis was performed using "SPSS" Statistical Analysis System Software. The obtained data were analyzed through the use of the analysis of variance (ANOVA). Comparison among each two groups was performed using the t-test. The significant differences were defined as P < 0.05.

## Results

### Hormonal assay

Animals underwent ovariectomy demonstrated decreased serum levels of T3 and T4 with increase of TSH levels when compared to the control and estradiol treated groups. Animals treated with estradiol demonstrated highly statistically significant increased levels of T3, T4 and decrease in TSH levels when compared to both control and ovariectomized groups (table 1).

**Table 1:** changes in the mean serum levels of thyroid hormones T3, T4 and TSH in the different groups

Mean ± SD	Group 1 (sham operated)	Group 2 (ovariectomized)	Group 3 (estradiol treated)
T3 ng/dL	54.001 ± 2.29	52.754 ± 2.14	65.007 ± 4.79*^#^

T4 ng/dL	66.021 ± 1.32	63.592 ± 1.46	80.615 ± 3.61*^#^

TSH microU/dL	33.96 ± 1.86	36.161 ± 1.46	24.587 ± 3.2*^#^

Marked differences * are significant at p < .05 compared to Group 1 (sham operated)

Marked differences # are significant at p < .05 compared to Group 2 (ovariectomized)

### Light microscopic results

**Figure 1:**
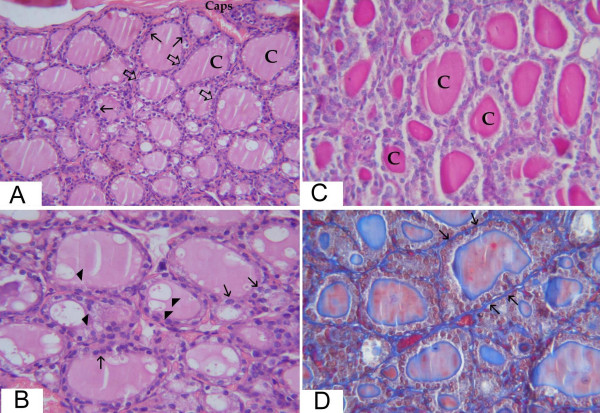
**light microscopic pictures of thyroid gland from sham operated rats**. (a) Large peripheral thyroid follicles (thick arrows) under the capsule (caps) lined with flattened and cuboidal epithelial lining (thin arrows) filled with colloid (C). The central follicles appear smaller in size (Hx&E × 200). (b) Higher magnification of the central zone from the previous section. The epithelial lining of the follicles is cuboidal (thin arrows) with rounded central or basal nuclei. The follicles are variable in size. The colloid contains peripheral vacuolization (arrow heads) (Hx&E × 400). (c) The central zone of thyroid of sham-operated rats showing variable amount of colloid filling the lumina of the follicles (C) (PAS × 400). (d) There is minimal amount of connective tissue in between the follicles (thin arrows) (Masson trichrome × 400).

In the sham-operated group, the thyroid gland was found to be composed of thyroid follicles which appeared generally oval or rounded lined with a single layer of cells. The central follicles appeared smaller in size than peripheral ones. The central follicles were lined with cuboidal cells with rounded nuclei, while the epithelial lining of the peripheral follicles was formed of flattened or low cuboidal cells. The amount of colloid substance varied from one follicular lumen to another. It showed different staining affinity with PAS. Some follicles contained conspicuous peripheral vacuoles. The connective tissue capsule was thin and the connective tissue septa were hardly identified dividing the gland into numerous small ill-defined lobules (fig. 1a-d).

**Figure 2:**
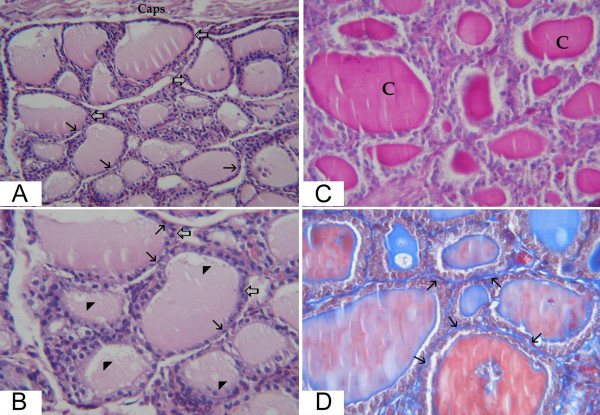
**light microscopic pictures of thyroid gland of group II (ovarictomized rat)**. (a) large peripheral thyroid follicles (thick arrows) beneath the capsule (caps). The central ones are variable in size with cuboidal and flattened epithelial lining (thin arrows) (Hx&E × 200). (b) higher magnification of the central zone from the previous section showing enlarged follicles (thick arrows) with flat epithelial lining and flattened nuclei of many cells (thin arrows). The colloid demonstrates minimal vacuolization (arrow heads) (Hx&E × 400). (c) follicles distended with large amount of colloid (C) (PAS × 400). (d) increased connective tissue in between the thyroid follicles (thin arrows) (Masson trichrome × 400).

In the ovariectomized rats, preserved secretory activity of the thyroid gland was noticed, it was mainly characterized by central follicles of a flattened, cuboidal or low prismatic epithelium surrounding the colloid. Occasionally, enlarged distended follicles with very flat epithelium were noticed. The peripheral follicles were large and lined with flattened epithelium. The colloid appeared filling up the entire lumen in most of the follicles with no or little peripheral vacuolization. The connective tissue around the follicles was more prominent than in sham-operated group (fig. 2a-d).

**Figure 3:**
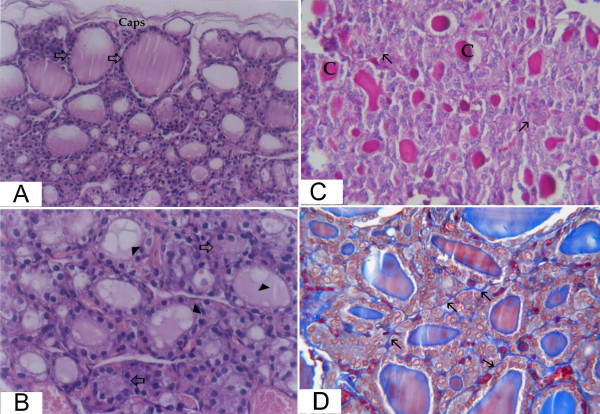
**light microscopic pictures of thyroid gland of group III (ovarictomized rat treated with estradiol)**. (a) The peripheral follicles (thick arrows) under the capsule (caps) are relatively larger than the central follicles (Hx&E × 200). (b) Higher magnification of the central zone showing very small follicles. Some follicles have no apparent lumina (thick arrows), while others contain absent or highly vacuolated colloid material (arrow heads) (Hx&E × 400). (c) There is little amount of the colloid in some follicles (C) while others demonstrate absent colloid (thin arrows) (PAS × 400). (d) There is minimal connective tissue (thin arrows) surrounding the thyroid follicles (Masson trichrome × 400).

The thyroid gland of estradiol treated group demonstrated very small follicles in the central region with high columnar or pyramidal epithelial lining surrounding narrow lumen. There were areas of disorganized follicles with complete obstruction of their lumina. The peripheral follicles were larger. The colloid was faintly stained and demonstrated extensive vacuolization. Connective tissue was minimal between the follicles (fig. 3a-d).

### Morphometric analysis of light microscopic results

In ovariectomized group, epithelial height was significantly decreased while the follicular area, colloidal area percent and connective tissue content were significantly increased when compared to sham-operated group.

In estradiol treated group, there was marked increase in epithelial height and decreased follicular area and colloidal area percent when compared to the other two groups. Connective tissue content was more or less similar to the sham-operated group. These results were summarized in (table 2).

**Table 2:** the statistical analysis of the morphometric data obtained by the image analysis

Mean ± SD	Group 1 (sham operated)	Group 2 (ovariectomized)	Group 3 (estradiol treated)
Epithelial height	9.05 ± 0.83	7.577 ± 0.9*	11.306 ± 0.83*^#^

Follicular area	3104.15 ± 216	4485.01 ± 577*	1841.78 ± 146*^#^

Colloidal area %	18.158 ± 1.34	23.958 ± 1.53*	8.16 ± 0.71*^#^

Connective tissue area %	1.36544 ± 1.24^#^	4.72668 ± 0.43*	1.3625 ± 0.19^#^

Marked differences * are significant at p < .05 compared to Group 1 (sham operated)

Marked differences # are significant at p < .05 compared to Group 2 (ovariectomized)

### Electron microscopic results

**Figure 4:**
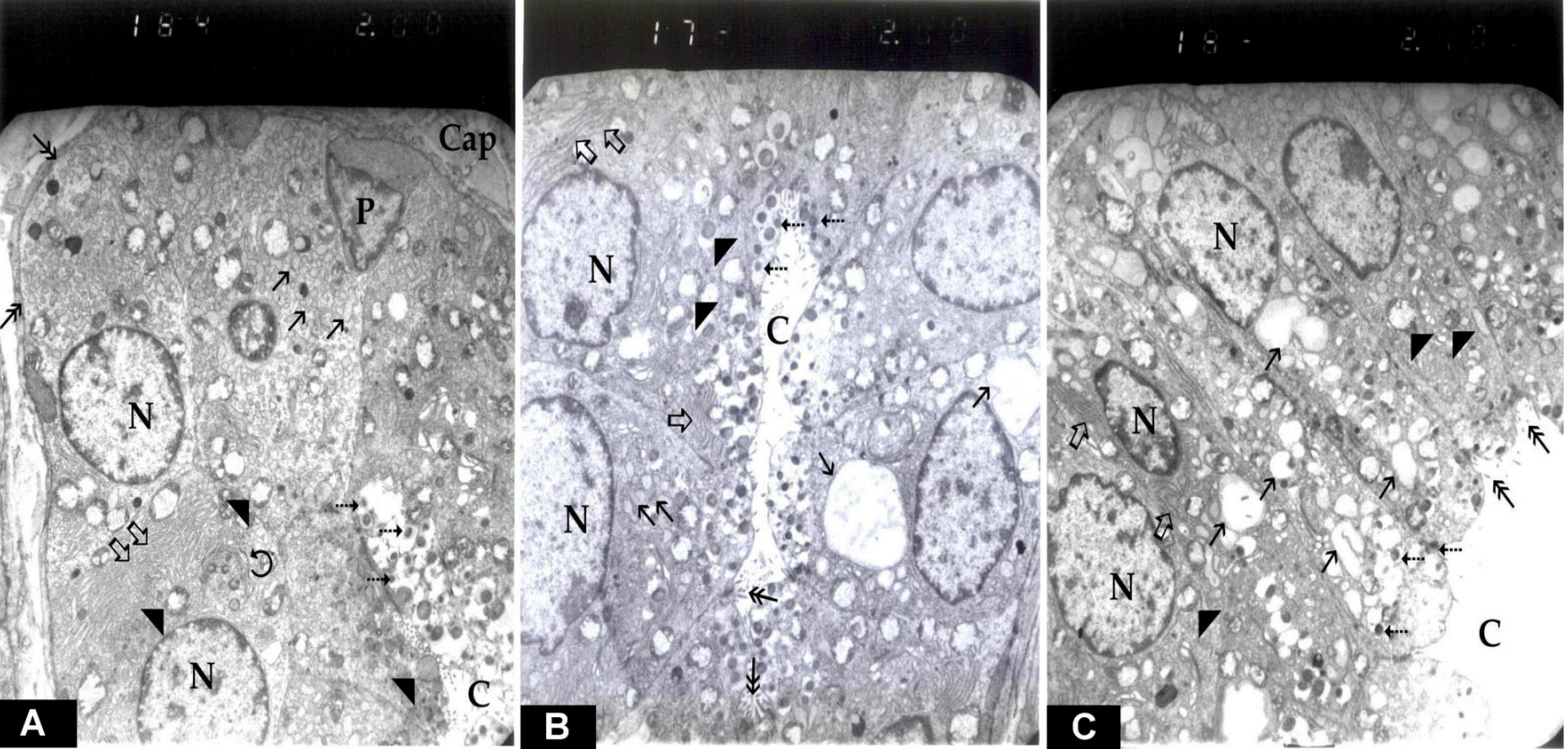
**electron micrograph of thyroid follicular cells in (a) sham operated rats, (b) ovariectomized rats and (c) ovariectomized rats treated with estradiol**. (a) This is a part of a thyroid follicle. The follicles are bounded with a basement membrane (double arrows) and surrounded with capillaries (Cap). Two follicular cells with rounded nuclei (N) and a parafollicullar cell (P) could be demonstrated. In the follicular cells; saccules of Golgi apparatus (curved arrow), prominent endoplasmic reticular cisternae (thick arrows), rounded mitochondria (arrow heads), lysosomes (dashed arrows) intracytoplasmic colloid vesicles (thin arrows) with mild apical vacuolization near the center of the follicle (C) could be demonstrated (EM × 2000). (b) There are rounded nuclei of follicular cells (N), while the mitochondria (arrow heads) and endoplasmic reticular cisternae (thick arrows) showed no remarkable changes. Intracytoplasmic colloid vesicles (thin arrows) were more prominent while the apex of the cell facing colloid (C) showed microvilli (double arrows) and many lysosomes (dashed arrows) with apical vacuolization could be clearly demonstrated (EM × 2000). (c) There are oval nuclei (N), endoplasmic reticular cisternae (thick arrows) and prominent rounded mitochondria (arrow heads) were comparable to the control group. Large amount of intracytoplasmic colloid vesicles (thin arrows) were much more evident, while the apex of the cell facing colloid (C) showed microvilli (double arrows) with much more apical vacuolization and many lysosomes (dashed arrows) (EM × 2000).

Electron microscopic study of the thyroid follicles in control sham operated rats showed rounded follicles formed of cuboidal cells with rounded nuclei. The follicles are bounded with a basement membrane and surrounded with capillaries. The cells are rich in rough endoplasmic reticulum and mitochondria in addition to a prominent Golgi apparatus and intracytoplasmic colloid vesicles. The apex of the cells adjacent to the follicular lumen showed apical cytoplasmic vacuolization with the presence of many lysosomes (fig. 4a).

Electron microscopic study of the thyroid follicles in ovariectomized rats showed thyroid follicles with cuboidal cells with rounded nuclei. Intracytoplasmic colloid vesicles were more prominent with well developed Golgi apparatus. In addition to that the apical cytoplasmic vacuolization and lysosomes could be demonstrated (fig 4b).

Electron microscopic study of the thyroid follicles in ovariectomized rats treated with estradiol showed increased activity of thyroid follicular columnar cells with oval nuclei and intracytoplasmic colloid vesicles which were much more prominent with clear apical cytoplasmic vacuolization (fig. 4c).

## Discussion

The end of the reproductive lifespan, in females of many species, is associated with hypoestrogenic state that lead to other physiological changes with increased risk of osteoporosis and cardiovascular diseases. Estrogen replacement therapy was accepted for prevention and treatment of human menopausal problems [16]. Unfortunately, recent evidence has connected hormonal treatment with undesirable side effects especially as a carcinogenic stimulator of cell proliferation and inducing genetic damage [17].

The main goal of the present study was to elucidate the effects of chronic estradiol treatment on thyroid gland structure and function in ovariectomized rat model, in doses comparable with those used in human therapy.

In the present study, ovariectomized rats demonstrated a decrease in the levels of T3 and T4 with an increase in the level of TSH when compared to animals with intact ovaries. A recent study by De Araujo et al. [7], demonstrated similar findings in ovariectomized rats. Also high levels of TSH were recorded in postmenopausal women in several studies [2,18]. While Sosic-Jurjevic et al. [19] reported increased levels of T3 and T4 this difference may be due to a shorter duration of estrogen administration.

In the present study, serum thyroid hormone levels seem to reflect the histological and morphometric changes in thyroid glands. In ovariectomized rats; the follicles were lined with low prismatic, cuboidal or flattened epithelium with significant decrease in epithelial height. The follicular area was increased as well as colloidal area percent. These findings indicated hypoactivity of the thyroid gland [20].

Estrogen has antifibrotic effects through the inhibition of key profibrotic factors, and through attenuation of fibroblast differentiation [21]. So the increased connective tissue content shown in this study in ovariectomized rats could be due to decreased estrogen antifibrotic effects as a result of estrogen withdrawal.

In our study electron microscopic examination of the thyroid gland from ovariectomized rats showed the cuboidal follicular cells with increased amount of intracytoplasmic colloid vesicles. While with estradiol treatment in ovariectomized rats, the follicular cells appeared columnar with oval nuclei with much more prominent intracytoplasmic colloid vesicles and clear apical vacuolization.

Zayed et al. [22] also reported decreased follicular colloid with increased intracytoplasmic colloid vesicles due to decreased activity of thyroid gland after ovariectomy with increased intracytoplasmic vesicles stored later as colloid in the follicular lumen which became wider.

Meanwhile, some reports have shown that the effects of estrogens on the thyroid gland may be indirect through the pituitary-thyroid axis [18] or as a consequence of increased serum levels of thyroxin binding globulin and the associated changes in thyroid function [23].

Taken together, the present results demonstrate that the thyroid glands of rats remain susceptible to the influence of ovariectomy and estrogen. The growth stimulatory effect of estrogen on benign and malignant human thyroid cells support the epidemiological data showing higher prevalence of thyroid carcinomas in females [24].

Although the extrapolation of these results to human should be interpreted with caution, it is highly conceivable that monitoring of thyroid function may be important for patients who are under or going to be under estrogen therapy.

## Conclusion

Decreased amount of estrogen may lead to thyroid hypofunction while estradiol treatment may lead to hyperactivity so it should be used very cautiously in the treatment of postmenopausal symptoms to avoid its undesirable stimulatory effect on the thyroid.

## Competing interests

The authors declare that they have no competing interests.

## Authors' contributions

All authors have made substantial contributions to design of the work, in addition to analysis and interpretation of data; and have been involved in drafting the article and revising it critically for important intellectual content; and have given final approval of the version to be published.
